# Effects of farrowing induction using cloprostenol on sow farrowing characteristics

**DOI:** 10.14202/vetworld.2022.1535-1540

**Published:** 2022-06-25

**Authors:** Nguyen Hoai Nam, Bui Tran Anh Dao, Peerapol Sukon

**Affiliations:** 1 Department of Animal Surgery and Theriogenology, Faculty of Veterinary Medicine, Vietnam National University of Agriculture, Trauqui, Gialam, Hanoi, Vietnam; 2 Department of Pathology, Faculty of Veterinary Medicine, Vietnam National University of Agriculture, Trauqui, Gialam, Hanoi, Vietnam; 3 Department of Anatomy, Faculty of Veterinary Medicine, Khon Kaen University, Khon Kaen, Thailand; 4 Research Group for Animal Health Technology, Khon Kaen University, Khon Kaen, Thailand

**Keywords:** birth interval, cloprostenol, dystocia, stillbirth

## Abstract

**Background and Aim:**

Previous findings regarding the effects of farrowing induction on the farrowing characteristics of sows are controversial. This study aimed to investigate the effects of farrowing induction on the following characteristics: (1) Proportion of sows that farrowed during working hours, (2) stillbirth rate, (3) number of stillbirths per farrow, (4) dystocia rate per farrow, (5) dystocia rate (the proportion of farrowings that had at least one dystocia event), (6) number of dystocia events per farrow, (6) farrowing duration, (7) birth interval, and (8) birth weight.

**Materials and Methods:**

Thirty-eight Landrace x Yorkshire sows were randomly allocated into two groups; the control group and the treatment group. In the control group (n = 18), sows farrowed spontaneously. In the treatment group (n = 20), farrowing was induced approximately 2 days earlier than the herd’s average length of gestation (7:00 am on day 114) by injecting cloprostenol into the perivulval region. All sows were supervised throughout their farrowing. We recorded the interval between induction and farrowing; total number of births; number of live, stillborn, and mummified piglet births; number of dystocia events; birth interval; farrowing duration; and birth weight. A generalized linear mixed model, a linear mixed-effects model, the Chi-squared test, and Student’s t-test were used to compare outcomes between the two groups.

**Results:**

Farrowing induction did not influence the percentage of sows that farrowed during working hours (7 am–5 pm), stillbirth rate, birth weight, and number of dystocia events per farrow. Farrowing induction led to an increase in birth interval, dystocia rate, dystocia per farrow (p < 0.05) and in addition to the percentage of sows that farrowed on the day following induction (60% vs. 27.8%; p < 0.05).

**Conclusion:**

Farrowing induction using a single dose of cloprostenol 2 days before the expected farrowing date can be performed with care to concentrate farrowing into a short interval. This can enhance the optimization of cross-fostering and the practice of an all-in-all-out strategy in the swine breeding industry.

## Introduction

The swine breeding industry has witnessed tremendous achievements following an increase in the number of total births, live births, litters per sow per year, and piglets weaned per sow per year, which is attributable to genetic selection, nutrition science, and breeding management [1]. Breeding management is a quick and effective tool for improving the reproductive performance of sows. If sows farrow during working hours, the rate of supervision is increased, and most newborns receive care immediately after birth, which reduces mortality, allows for the optimization of cross-fostering, and reduces weaning age variability [2]. However, because sows usually farrow at night [3], supervision is difficult on a commercial scale, which affects breeding management.

Farrowing induction in pigs has shown promising results in both research and production settings. The previous studies have reported that farrowing induction using prostaglandin F2a (PGF2α) or its analogs increases the predictability of parturition time [4] and the proportion of sows that farrow during working hours (7 am–5 pm) [3, 5–7]. Furthermore, farrowing induction did not affect the stillbirth rate or birth weight when it was performed 1–2 days before the expected farrowing date [8]. Prostaglandin products have been used in combination with uterotonic drugs, such as oxytocin and carbetocin, to shorten the interval between induction and farrowing [3, 5, 6, 9]. However, there are concerns regarding the harmful effects of oxytocin and carbetocin on the rates of stillbirth and dystocia [10]. A meta-analysis by Muro *et al*. [11] revealed that the stillbirth rate increased by 30% with oxytocin, while the need for farrowing assistance increased by 130% with oxytocin and by 40% with carbetocin. However, only a few studies have investigated the effect of farrowing induction on dystocia [8]. Therefore, more studies are needed to investigate the effect of farrowing induction on this trait.

This study aimed to evaluate the effects of farrowing induction on the proportion of sows farrowing during working time, stillbirth rate, number of stillbirths per farrow, farrowing duration, birth interval, birth weight, dystocia rate per farrow, dystocia rate, and number of dystocia events per farrow using a single dose of cloprostenol administered on day 114 of gestation (about 2 days before the herd’s average gestation length).

## Materials and Methods

### Ethical approval

The present experiment was reviewed and approved by the Committee on Animal Research and Ethics, Faculty of Veterinary Medicine, Vietnam National University of Agriculture (CARE-2021/03).

### Study period and location

The study was conducted from March to May 2021 at a commercial swine farm in North Vietnam with a breeding capacity of 500 sows.

### Animals, diet, and experimental design

We included 38 Landrace x Yorkshire sows with good body condition scores and average parity of 3.9 ± 1.4 (range, 2–5). The sows, in estrus, were inseminated with Duroc boar semen (day 0 of gestation).

The sows were vaccinated against classical swine fever, Aujeszky’s disease, foot and mouth disease, porcine reproductive and respiratory syndrome, and porcine parvovirus. They were bathed once or twice per day, depending on the ambient temperature. During the study period, average 24-hour temperature ranged between 20.2°C and 27.3°C. Gestating sows received 2.0–3.0 kg of commercial feed per day until day 111, after which they received 1 kg of feed per day until farrowing. On the day of farrowing, the sows received no feed. Water was provided to sows *ad libitum* through a nipple system. The gestating sows were housed in individual gestation crates of 1.32 m^2^ area. At around 7 days before the expected farrowing date, the sows were moved to individual farrowing pens of 3.96 m^2^ area. The farrowing pens were fully slatted with a concrete floor. Sows were retained within the 1.32 m^2^ sow area, centered within the pen. A creep area at both sides of the sow area was covered with hard plastic slats. For newborn piglets (in their 1^st^ week), a 0.6 m^2^ creep area was heated at one corner with an infrared lamp.

The sows were stratified by parity and randomly divided into two groups, that is, control group and treatment group. The control group consisted of 18 sows that spontaneously farrowed. The treatment group consisted of 20 sows in which farrowing was induced by injecting 175 mg cloprostenol (Han-prost; Hanvet, Hanoi, Vietnam) into the perivulva region at 7 am on day 114 of gestation, which was approximately 2 days before the herd’s expected farrowing date. All sows in both groups were continuously monitored; their farrowing was fully supervised from the birth of the first piglet to the birth of the last piglet. The sows were intramuscularly injected with amoxicillin trihydrate (15 mg/kg) (Amoxisol® L.A, 15 mg/kg, Bayer, Vietnam) after the first birth; they were treated with at least three doses of amoxicillin, where the injection was given on a 48 h interval. To hasten fetal membrane expulsion, exogenous oxytocin was administered following the last birth. After farrowing, the sows’ feed was increased to an *ad libitum* level, which was commonly reached on day 6 postpartum. During the lactation period, the sows were fed 3 times per day (7:00 am, 10:00 am, and 3:00 pm).

### Data collection

During farrowing, the sows were supervised by at least two veterinarians. The total number of births (live, stillborn, and mummified piglets), birth interval, farrowing duration, dystocia, and birth weight were recorded. Stillborn piglets were those born dead without signs of autolysis. Mummified piglets were those born dead with signs of autolysis and mummification. Birth interval was defined as the interval between the births of two successive piglets. Farrowing duration was defined as the interval between the birth of the first piglet and the birth of the last piglet. Dystocia was recorded when a birth interval exceeded 45 min [12, 13]. Birth assistance was performed only when a dystocia event occurred. Because of the farm’s regulations restricting birth assistance, manual assistance was not provided in all cases of dystocia; therefore, the rate of birth assistance was not recorded. Birth weight was measured at 5 min after birth using a digital scale with 5 g precision.

The stillbirth rate, number of stillbirths per farrow, number of mummified fetuses per farrow, dystocia rate per farrow, dystocia rate, and number of dystocia events per farrow were calculated. The stillbirth rate was calculated as the proportion of piglets born as stillbirths. The dystocia rate per farrow was defined as the proportion of piglets born through a dystocia event. The dystocia rate was defined as the proportion of farrowings that had at least one dystocia event.

### Statistical analysis

Gestation length, number of births per farrow (live piglets, stillborn piglets, and mummified fetuses), number of dystocia events per farrow, and farrowing duration were compared between the treatment and control groups using Student’s t-test. Birth weight and birth interval were compared between the two groups using a linear mixed-effect model (LMEM). Stillbirth rate and dystocia rate per farrow were compared between the two groups using a generalized linear mixed model (GLMM). In both the LMEM and GLMM models, the sow was treated as a random factor to deal with hierarchical data where piglets were nested within sows. Dystocia rate, proportion of sows that farrowed on the day following induction, and proportion of sows that farrowed during working hours were compared between the two groups using a Chi-square test. p < 0.05 was considered statistically significant. The t-test and Chi-square test were performed using Statistical Package for the Social Sciences software version 20.0 (IBM, Armonk, NY, USA). The LMEM and GLMM analyses were performed using the R Studio Desktop ver. 1.3.1093 (R Foundation for Statistical Computing, Vienna, Austria).

## Results

The mean interval between induction and farrowing was 29.5 ± 14.9 h (range, 12–77 h). The numbers of induced sows that farrowed on days 114, 115, 116, and 117 were 5/20 (25%), 12/20 (60%), 2/20 (10%), and 1/20 (5%), respectively. In the control group, the numbers of sows that farrowed on days 114, 115, 116, 117, and 118 were 3/18 (16.7%), 5/18 (27.8%), 5/18 (27.8%), 3/18 (16.7%), and 2/18 (11.0%) (table not shown).

A comparison of different farrowing characteristics between the treatment and control groups is shown in Table-1. The length of gestation in induced sows was significantly shorter than that in the control group (115.0 vs. 115.8 days, p = 0.018), and the birth interval in the treatment group was significantly longer than that in the control group (21.8 vs. 15.0 min, p = 0.032). Compared with the control group, the induced group showed increases in the dystocia rate per farrow (11.5 vs. 6.3, p = 0.048), dystocia rate (90% vs. 50%, p = 0.007), and proportion of sows that farrowed on the day following induction (60% vs. 27.8%, p = 0.046). However, farrowing induction did not influence the number of live births per farrow (12.8 vs. 13.7, p = 0.355), number of stillbirth per farrow (0.75 vs. 1.11, p = 0.429), number of mummified fetuses per farrow (0.45 vs. 0.22, p = 0.269), number of dystocia events per farrow (1.5 vs. 0.9, p = 0.122), proportion of sows that farrowed during working hours (55% vs. 50%, p = 0.758), stillbirth rate (6.8% vs. 7.4%, *p* = 0.776), farrowing duration (283.5 vs. 209.6 min, p = 0.107), and birth weight (147.0 vs. 1436.3 g, p = 0.791).

**Table 1:** Comparison of different parameters between farrowing induction and control groups.

Parameters	Treatment	Control	p-value
Gestation length (day)	115.0 ± 0.8 (n = 20)	115.8 ± 1.3 (n = 18)	0.018*
Total born	14.0 ± 4.1 (n = 280)	15.0 ± 3.1 (n = 270)	0.384*
Number of born alive piglets per farrow	12.8 ± 3.5 (n = 255)	13.7 ± 2.3 (n = 246)	0.355*
Number of stillbirths per farrow	0.75 ± 1.11 (n = 15)	1.11 ± 1.64 (n = 20)	0.429*
Number of mummified fetuses per farrow	0.45 ± 0.76 (n = 9)	0.22 ± 0.43 (n = 4)	0.269*
Number of dystocia events per farrow	1.5 ± 0.9 (n = 30)	0.9 ± 1.2 (n = 16)	0.122*
Farrowing duration (min)	283.5 ± 161.0 (n = 20)	209.6 ± 105.3 (n = 18)	0.107*
Birth weight (g)	1417.0 ± 350.1 (n = 266)	1436.3 ± 653.9 (n = 263)	0.791**
Birth interval (min)	21.8 ± 40.0 (n = 260)	15.0 ± 22.0 (n = 252)	0.032**
Sows farrowing during working time (%)	55 (11/20)	50 (9/18)	0.758***
Sows farrowing on the following day of induction (%)	60 (12/20)	27.8 (5/18)	0.046***
Dystocia rate (%)	90 (18/20)	50 (9/18)	0.007***
Dystocia rate per farrow (%)	11.5 (30/260)	6.3 (16/252)	0.048****
Stillbirth rate (%)	6.8 (19/280)	7.4 (20/270)	0.853****

The results were presented in mean ± standard deviation or percentage. Dystocia rate was calculated as the proportion of sows farrowed with at least one dystocic event. Dystocia rate per farrow was calculated as proportion of piglets born with a dystocic event. *, **, ***, and **** meant that the comparison was done using t-test, linear mixed effect model, Chi-square test, and generalized linear mixed model, respectively

## Discussion

Farrowing induction has been used to increase the proportion of sows that farrow during working hours [14], which increases the rate of farrowing supervision. We found that the proportion of sows farrowed during working hours remained unchanged by farrowing induction. This finding agrees with a study by Tospitakkul *et al*. [5], which reported that 56.5% of induced sows farrowed during working hours compared with 50.0% in a control group. Kirkwood and Aherne reported that 56% of induced sows farrowed during working hours when induced with a single dose of cloprostenol [4]. Despite a failure in increasing the proportion of sows farrowing during working hours, induction increased the percentage of sows that farrowed on day 115 after injection of cloprostenol (50%). In the control group, the numbers of farrowings across days 114, 115, 116, and 117 were equally distributed (16.7–27.8%). Although induction was unable to increase the percentage of sows farrowing during working hours, it concentrated the farrowings into a shorter interval. This allowed optimization of piglet cross-fostering and the practice of an all*-*in all*-*out strategy in the swine breeding industry.

Farrowing induction has been found to increase farrowing duration [5, 15] and birth interval [5] in some studies, while other studies have not observed similar results [3, 16–20]. This discrepancy may arise due to differences in the interval between the dates of induction and expected farrowing; farrowing duration may increase due to induction of farrowing 2–3 days before the expected farrowing date [15]. In our study, induction was performed approximately 2 days before the expected farrowing date, which was expected to increase farrowing duration. However, a significant change was not observed because of the small sample size despite a trend toward longer farrowing duration in induced sows. Early farrowing induction may impair farrowing duration and birth interval through endocrinological mechanisms of parturition.

In spontaneously farrowing sows, relaxin peaks at around 1 day before parturition [21]. The injection of PGF2α induces relaxin secretion [22]. More than a half of PGF2α analog-induced sows have two peaks of relaxin; the first peak occurs approximately 1 h after PGF2α administration and the second peak occurs closer to the time of farrowing [23]. Relaxin dilates the uterine cervix [24, 25] and inhibits the secretion of oxytocin [26]. Sows with two peaks of relaxin have higher concentrations of relaxin than sows with one peak of relaxin. Thus, farrowing duration is longer in sows with two peaks of relaxin than that in sows with one peak of relaxin [23]. In this study, cloprostenol was administered to the sows approximately 2 days before the expected farrowing date, which may have caused the second peak of relaxin. This potential increase in relaxin secretion might have impaired oxytocin secretion, resulting in longer farrowing duration and birth intervals in the treatment group than those in the control group. Moreover, many other hormones, including estrogen, progesterone, oxytocin, and cortisol were found to influence the farrowing process [21, 27, 28]. In our study, early induction might have caused changes in hormone production that impaired farrowing kinetics, resulting in longer farrowing duration and birth intervals. To observe hormonal changes and their effects on farrowing characteristics, future studies should measure the levels of cortisol, estradiol, progesterone, relaxin, and other hormones in sows induced on different days relative to the expected farrowing date.

We found that farrowing induction on day 114 of gestation did not influence the birth weight of piglets. This finding agrees with the previous studies [5–7, 16–18, 20, 29–31]. However, some studies have reported that birth weights were reduced in induced sows compared with controls [9, 32, 33]. However, reduced birth weights in induced sows might be due to early induction (≥3 days before the expected farrowing dates) [32, 33] or natural differences among groups [9]. Our finding that farrowing induction had no significant effect on birth weight agrees with the finding of a meta-analysis by Monteiro *et al*. [8], which concluded that birth weight decreases only when induction is performed ≥3 days before the expected farrowing date.

Some studies reported that farrowing induction reduced the rate of stillbirths [34–36]. These findings may have been influenced by naturally heavier piglets born to induced sows [34, 36] or by a 100% farrowing supervision rate in the induced group, compared with non-supervision in the control group [35]. Previous studies [37, 38] found that stillbirth rates increased when farrowing induction was performed on day 111 of gestation, which is also associated with reduced birth weight and an increased rate of premature births. An increased stillbirth rate was reported with farrowing induction on days 113–115; however, it appeared to be due to altrenogest supplementation approximately 7 days before induction rather than from induction itself [39]. Our present results corroborate the findings of many previous studies that found no effect of farrowing induction on the stillbirth rate [3, 6, 9, 16–18, 32, 33, 40, 41]. A meta-analysis by Monteiro *et al*. [8] concluded that induction 2–3 days before the expected farrowing date did not influence the stillbirth rate. In the present study, induction was performed approximately 2 days before the expected farrowing date to avoid premature birth. The birth weight of piglets and the stillbirth rate were found to be nearly identical between the treatment and control groups.

Most studies investigating the effects of induction on dystocia and the need for birth assistance have reported non-significant findings [9, 17, 31, 38]. In a study by Chantaraprateep *et al*. [31], the findings may have been influenced by the small sample sizes (n = 10 in each group), as the dystocia rate among groups ranged between 0% and 50%. A study by Decaluwé *et al*. [17] did not report on the dystocia rate, dystocia rate per farrow, or number of dystocia events per farrow. The increased dystocia rate in this study corroborated the finding of Boonraungrod *et al*. [3]; the dystocia rate in the control group was 33.8%, whereas the dystocia rates in two induced groups were 50% and 73.6%. Another study found that double administration of PGF2α reduced the dystocia rate per farrow (2.7% vs. 10.6%), but single administration failed to obtain similar results (11.6% vs. 10.6%); the dystocia rates were 53.6%, 26.7%, and 41.7% in the single-dose, double-dose, and control groups, respectively [5]. In the present study, we found that both the dystocia rate and the dystocia rate per farrow in the treatment group were higher than those in the control group. To the best of our knowledge, this is the first study to report an increase in the dystocia rate per farrow due to farrowing induction. As previously discussed, farrowing induction used in this study might have affected the production of hormones, impairing farrowing kinetics and thereby increasing dystocia rates.

## Conclusion

Farrowing induction using a single dose of cloprostenol approximately 2 days before the expected farrowing date failed to increase the proportion of sows farrowing during working hours. However, it increased the percentage of sows farrowing on the day following induction. Farrowing induction increased the dystocia rate, dystocia rate per farrow, and birth interval. Induction potentially increased farrowing duration but did not affect either birth weight or stillbirth rate. Our results indicate that farrowing induction can be used to increase the proportion of sows that farrow on the day following induction. This allows optimization of cross-fostering and implementation of the all-in-all-out strategy. Farrowing induction should be practiced with care because it may increase dystocia. Future studies are needed to validate this effect and investigate other hormonal changes following induction.

## Authors’ Contributions

NHN: Collected the data. NHN, PS, and BTAD: Conceived and designed the study, analyzed data, interpreted results, and wrote the manuscript. All authors have read and approved the final manuscript.
